# A Submillimeter-Level Relative Navigation Technology for Spacecraft Formation Flying in Highly Elliptical Orbit

**DOI:** 10.3390/s20226524

**Published:** 2020-11-15

**Authors:** Xiaoliang Wang, Deren Gong, Yifei Jiang, Qiankun Mo, Zeyu Kang, Qiang Shen, Shufan Wu, Dengfeng Wang

**Affiliations:** 1 School of Aeronautics and Astronautics, Shanghai Jiao Tong University, Shanghai 200240, China; xlwang12321@sjtu.edu.cn (X.W.); jamesjiang@sjtu.edu.cn (Y.J.); moqk@163.com (Q.M.); kangzeyu@sjtu.edu.cn (Z.K.); qiangshen@sjtu.edu.cn (Q.S.); shufan.wu@sjtu.edu.cn (S.W.); 2 Institute of Space Radio Technology, Xi’an 710100, China; dfwang_aero@163.com

**Keywords:** spacecraft formation flying, highly elliptical orbit, microwave ranging, relative navigation

## Abstract

Spacecraft formation flying (SFF) in highly elliptical orbit (HEO) has attracted a great deal of attention in many space exploration applications, while precise guidance, navigation, and control (GNC) technology—especially precise ranging—are the basis of success for such SFF missions. In this paper, we introduce a novel K-band microwave ranging (MWR) equipment for the on-orbit verification of submillimeter-level precise ranging technology in future HEO SFF missions. The ranging technique is a synchronous dual one-way ranging (DOWR) microwave phase accumulation system, which achieved a ranging accuracy of tens of microns in the laboratory environment. The detailed design and development process of the MWR equipment are provided, ranging error sources are analyzed, and relative orbit dynamic models for HEO formation scenes are given with real perturbations considered. Moreover, an adaptive Kalman filter algorithm is introduced for SFF relative navigation design, incorporating process noise uncertainty. The performance of SFF relative navigation while using MWR is tested in a hardware-in-the-loop (HIL) simulation system within a high-precision six degrees of freedom (6-DOF) moving platform. The final range estimation errors from MWR using the adaptive filter were less than 35 μm and 8.5 μm/s for range rate, demonstrating the promising accuracy for future HEO formation mission applications.

## 1. Introduction

Spacecraft formation flying (SFF) has attracted a great deal of attention, since it can perform space missions with more reliability, adaptability, and low life-cycle cost, compared with traditional monolithic spacecraft [1]. Several SFF missions have successfully been deployed in low Earth orbit (LEO), such as the Gravity Recovery and Climate Experiment (GRACE) mission for precise Earth gravity field measurement [2], EO-1/LandSat 7 for Earth observation [3], the TanDEM-X mission for the generation of high-precision digital elevation models by using a high-resolution interferometric synthetic-aperture radar [4,5], and PRISMA, for the on-orbit demonstration of millimeter-level SFF technology [6].

With the development of space technologies in recent decades, scientists have attempted to explore outer space far beyond Earth, which has turned their eyes toward highly elliptical orbit (HEO) SFF technologies. The most famous examples include the Magnetospheric Multiscale mission (MMS) launched by the National Aeronautics and Space Administration (NASA), a Solar Terrestrial Probes mission that studies the Earth’s magnetosphere [7]. The Cluster II mission directed by the European Space Agency (ESA) is aimed towards space physics observation [8]. These two HEO SFF missions are comprised of four instrumented spacecraft operating in an adjustable pyramid-like constellation, with verified guidance, navigation, and control (GNC) technology on orbit.

The realization of precise SFF GNC technologies requires the development of actuators, metrologies, and algorithms, where the metrologies system conducts the baseline of GNC, since it provides the direct relative-states measurement between the spacecrafts in formation. Typically, the metrologies system will cover both radio frequency (RF) and optical metrologies. The RF terminal receives signals from other spacecraft, and processes time-of-flight spread-spectrum code and carrier phase that allow for the retrieval of distance and line-of-sight (LOS) measurement with an accuracy of 1 cm (distance) and 1° (LOS). The optical metrologies can be conducted using geometric and interferometric concepts, achieving different ranges and accuracies. Optical position metrology for XEUS is reported as a coarse and fine sensor, which achieves accuracy of tens of microns, with a maximum relative distance of 120 m [9,10]. The dual-wavelength interferometer (DWI) was chosen as a fine longitudinal sensor for the DARWIN mission, with a ranging accuracy of about 10 μm within 250 m distance [11,12].

China’s space agency has recently approved an HEO SFF mission, called the Highly Elliptical Formation Flying Demonstration (HEFFD) mission, consisting of two spacecraft for science observation, quite similar to the Proba-3 mission [13,14]. The two formation spacecrafts are preliminarily designed in different formation configurations with a relative distance of several meters to hundreds of kilometers, according to the real task on orbit. Besides the original observation data obtained, the most anticipated reward of this mission is the design and development of technologies that enable future HEO SFF missions, especially for the on-orbit demonstration of high-precision GNC technology. The primary ranging payload is designed based on optical metrology, which delivers micron-level relative lateral/longitudinal ranging between spacecraft during the science observation period in apogee.

Moreover, a K-band microwave ranging (MWR) payload is developed particularly for this HEO formation mission for the following reasons:

- In addition to optical metrology, (a) MWR can provide precise ranging results as backup equipment, during the science observation period around apogee; and (b) can avoid sunlight disturbance which occurs in the optical spectrum, for specific space science missions such as solar corona observation.
- Microwave ranging relies on a carefully designed transceiver antenna with relatively wider main lobe angle of a few degrees, which greatly reduces the burden of the high-precision pointing mechanism used for optical metrology. At the same time, microwave ranging can operate in pseudo-code mode or carrier mode for coarse/precise ranging if necessary, as required by mission control, providing flexible solutions to the GNC system.
- We have inherited a great deal of microwave ranging technology from existing space experience. MWR has been developed and tested previously, and successfully verified in one LEO formation mission [15].
- As an essential supplement to the MWR system, BeiDou III B1C/B2a dual-frequency new-generation navigation signal receive and process technology can be fully tested and verified in HEO formation missions. The B1C/B2a dual-frequency receiver on-board can provide highly precise time synchronization solutions with nanosecond precision, which serves as a time-scale benchmark between formation spacecrafts. Moreover, the receiver can provide precise stand-alone navigation solutions while using a precise orbit determination algorithm.
- Microwave can also be used for real-time data transmission between spacecraft for original scientific data exchange, differential GPS measurement data transmission for relative navigation, etc.

This paper focuses on the principle, design, development, and testing of MWR equipment that will be deployed in the HEFFD mission. The following content is organized as follows: Section 2 introduces the development of the submillimeter-level accuracy microwave ranging technique and MWR equipment. Section 3 describes the relative orbit models and perturbations considered during real formation flight in space. Section 4 provides the relative navigation filter algorithm that deals with process noise uncertainty, and the simulation results using MWR and proposed methods are finally given in Section 5.

## 2. Submillimeter-Level Microwave Ranging

### 2.1. MWR Measurement Modeling

MWR measures the biased distance between the antenna phase centers of two satellites through microwave carrier phase accumulation and Doppler drift, and then derives the distance variation and its rate between two satellites. Basically, the whole system is a synchronous dual one-way ranging (DOWR) microwave phase accumulation ranging system [16]. Three techniques are adopted by MWR that ensure ranging accuracy:

- Correction of ionospheric influence by dual-frequency measurement during space flight around perigee;
- Synchronous bidirectional ranging comparison that cancels the long-term instability of the oscillator source; and,
- Carrier phase measurement by frequency difference to improve the accuracy.

**Figure 1:**
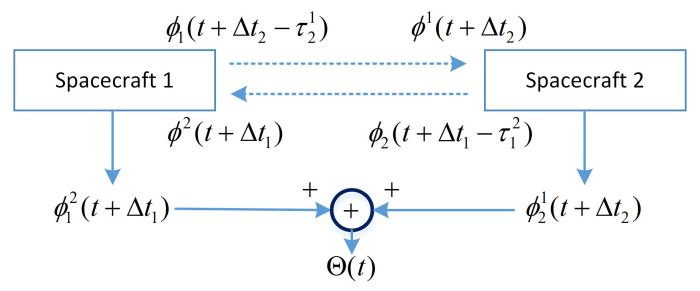
Principle of dual one-way ranging.

Figure 1 shows the principle of DOWR microwave phase measurement.

The dual-way phase measurement is defined as the summation of one-way ranging:

(1) $$\Theta \left(t\right)\equiv \phi_{1}^{2}\left(t+\Delta t_{1}\right)+\phi {}_{2}^{1}\left(t+\Delta t_{2}\right)$$

and the carrier phase measurement received by satellite *i* at time *t* can be modeled as:

(2) $$\begin{array}{c} \phi_{i}^{j}\left(t+\Delta t_{i}\right)=\phi_{i}\left(t+\Delta t_{i}\right)-\phi^{j}\left(t+\Delta t_{i}\right)+N_{i}^{j}+I_{i}^{j}+d_{i}^{j}+\varepsilon_{i}^{j}\\ i,j=1,2,\;i\neq j \end{array}$$

where $\phi_{i}\left(t+\Delta t_{i}\right)$ is the reference phase of satellite *i*, $\phi^{j}\left(t+\Delta t_{i}\right)$ is the received signal phase from satellite *j*, $\Delta t_{i}$ is the oscillator instability error, $N_{i}^{j}$ is the phase ambiguity, $I_{i}^{j}$ is the phase bias due to ionosphere delay, $d_{i}^{j}$ is the phase bias due to other reasons, and $\varepsilon_{i}^{j}$ is the phase bias due to measurement noise.

Substitute the receiving phase to sending phase

(3) $$\phi^{j}\left(t+\Delta t_{i}\right)=\phi_{j}\left(t+\Delta t_{i}-\tau_{i}^{j}\right)$$

where $\tau_{i}^{j}$ is the propagation time from satellite *j* to *i*.

Each phase $\phi_{i}\left(t\right)$ is the summation of reference phase ${\bar{\phi }}_{i}$ and phase error due to oscillator $\delta \phi_{i}$. Represent the phase rate ${\dot{\phi }}_{i}\left(t\right)$ as reference frequency ${\bar{f}}_{i}$. Finally, the DOWR can be written as:

(4) $$\begin{array}{cc} \Theta (t)= & \left({\bar{f}}_{1}\tau_{2}^{1}+{\bar{f}}_{2}\tau_{1}^{2}\right)\\ \; & +\{\left[\delta \phi_{1}\left(t\right)-\delta \phi_{1}\left(t-\tau_{2}^{1}\right)\right]+\left[\delta \phi_{2}\left(t\right)-\delta \phi_{2}\left(t-\tau_{1}^{2}\right)\right]\}\\ \; & +\left({\bar{f}}_{1}-{\bar{f}}_{2}\right)\left(\Delta t_{1}-\Delta t_{2}\right)\\ \; & +\left(\delta {\dot{\phi }}_{1}-\delta {\dot{\phi }}_{2}\right)\left(\Delta t_{1}-\Delta t_{2}^{}\right)+N+I+d+\varepsilon \end{array}$$

where ${\bar{f}}_{1}\tau_{2}^{1}+{\bar{f}}_{2}\tau_{1}^{2}$ denotes the real phase measurement;

$\left\{\left[\delta \phi_{1}\left(t\right)-\delta \phi_{1}\left(t-\tau_{2}^{1}\right)\right]+\left[\delta \phi_{2}\left(t\right)-\delta \phi_{2}\left(t-\tau_{1}^{2}\right)\right]\right\}$ denotes the phase error due to the oscillator, and the middle-term/long-term oscillator phase error can be eliminated if the propagation time is longer than one millisecond using DOWR;

$\left({\bar{f}}_{1}-{\bar{f}}_{2}\right)\left(\Delta t_{1}-\Delta t_{2}\right)$: time-tag error;

$\left(\delta {\dot{\phi }}_{1}-\delta {\dot{\phi }}_{2}\right)\left(\Delta t_{1}-\Delta t_{2}^{}\right)$: coupling term of oscillator and time tag;

N+I+d+ε: other errors; and,

$\left({\bar{f}}_{1}\tau_{2}^{1}+{\bar{f}}_{2}\tau_{1}^{2}\right)\approx \left({\bar{f}}_{1}+{\bar{f}}_{2}\right)\tau +\Delta \Theta_{TOF}\left(t\right)$, with time of flight (TOF) defined as τ=ρ(t)/c.

$\Delta \Theta_{TOF}\left(t\right)$: correction of TOF, which can be calibrated using a precise orbit determination technique.

Dual ranging R(t)≡λΘ(t), with

(5) $$\lambda =c/\left({\bar{f}}_{1}+{\bar{f}}_{2}\right)$$

Subsequently, we have

(6) $$R\left(t\right)=\rho \left(t\right)+\rho_{TOF}\left(t\right)+\rho_{err}\left(t\right)+N^{\prime }+I^{\prime }+d^{\prime }+\varepsilon^{\prime }$$

where ρ(t) is the instant distance, $\rho_{TOF}\left(t\right)$ is the correction of time of flight (TOF), $\rho_{err}\left(t\right)$ is the ranging error due to oscillator and time tag, $N_{i}^{j}$ is the ambiguity, $I^{\prime }$ is the ionosphere error, $d^{\prime }$ is the phase bias due to other reasons, and $\varepsilon^{\prime }$ is the ranging error due to measurement noise.

The one-way phase measurement of $\phi_{i}^{j}\left(t+\Delta t_{i}\right)$ is transferred to instant distance ρ(t), and DOWR ranging $R_{K}$&$R_{Ka}$ can be derived while using K&Ka band measurement. Finally, DOWR ranging without the influence of the ionosphere can be given as

(7) $$R=\frac{{\bar{f}}_{Ka}^{2}R_{Ka}-{\bar{f}}_{K}^{2}R_{K}}{{\bar{f}}_{Ka}^{2}-{\bar{f}}_{K}^{2}}$$

The principle of differential frequency phase measurement is to change the reference signal and measured signal into intermediate frequency (IF), or lower frequency, by mixing the radio frequency (RF) and local frequency, followed by a phase comparison. Because the IF signal entering the phase measurement system is much lower than the RF, the phase period is expanded, which greatly improves the phase measurement accuracy.

### 2.2. Ranging Error Sources Analysis

Carrier phase measurement errors are mainly caused by the following reasons: local oscillator phase noise, system noise, and dynamic stress error.

1. Carrier phase measurement error due to system noise
  The carrier phase measurement error due to system noise can be given as (1σ):
  (8) $$\sigma_{nois}=\frac{\lambda }{2\pi }\sqrt{\frac{B_{n}}{c/n_{0}\;}\left(1+\frac{1}{2Tc/n_{0}\;}\right)}\;\left(\mu m\right)$$
  where $B_{n}=2$ Hz, the noise bandwidth of the phase-locked loop (PLL); λ is the length of the carrier wave (K band $12.3\times 10^{3}$μm, Ka band $9.2\times 10^{3}$μm); T=0.02 s, the PLL pre-detection integration time; and $c/n_{0}=10^{65/10}$ is the carrier-to-noise ratio ($C/N_{0}=65$ dB-Hz).
  With calculation, the measurement errors due to system noise are $\sigma_{nois-K}=15.4$ μm(1σ), $\sigma_{nois-Ka}=11.5$ μm(1σ).
2. Carrier phase measurement error caused by phase noise of local oscillator
  The carrier phase measurement error (for the third-order tracking loop) introduced by the local oscillator’s Allan variance can be given by the following empirical formula:
  (9) $$\sigma_{Allan3}=160\cdot \frac{\lambda }{360}\cdot \frac{\sigma_{A}\left(\tau \right)f_{c}}{B_{n}}\;\left(\mu m\right)$$
  where $B_{n}=2$ Hz, the bandwidth of the PLL; $\tau =1/B_{n}=1$ s, the short-term stability length of the Allan variance; $\sigma_{A}\left(\tau \right)=3\times 10^{-13}$, the Allan variance; $f_{c}$ is the carrier frequency; K = 24.5 GHz; and Ka = 32.7 GHz.
  With calculation, the phase measurement errors due to local oscillator Allan variance are $\sigma_{Allan3-K}=20.1$ μm(1σ) for the K band and $\sigma_{Allan3-Ka}=15$ μm(1σ) for the Ka band.
3. Phase measurement error due to dynamic stress
  Dynamic stress error is closely related to the tracking loop order. For three-order PLL, the dynamic stress is given as:
  (10) $$\sigma_{PLL3}=0.4828\frac{d^{3}R/dt^{3}\;}{B_{n}^{3}}\;\left(\mu m\right)$$
  where $d^{3}R/dt^{3}$ is the line of sight (LOS) acceleration rate (μm/s${}^{3}$). For the PLL bandwidth Bn=2 Hz, the LOS acceleration rate is 10 μm/s${}^{3}$, and the dynamic error $\sigma_{PLL3}=0.60$ μm(1σ).
4. Summary of phase measurement error
  The total measurement error while using three-order PLL can be modeled as:
  K band: $\sigma_{\sum -K}=\sqrt{\sigma_{nois-K}^{2}+\sigma_{Allan3-K}^{2}+\sigma_{PLL3}^{2}}=\sqrt{15.4^{2}+20.1^{2}+0.6^{2}}=25.3$ (μm)
  Ka band: $\sigma_{\sum -Ka}=\sqrt{\sigma_{nois-Ka}^{2}+\sigma_{Allan3-Ka}^{2}+\sigma_{PLL3}^{2}}=\sqrt{11.5^{2}+15^{2}+0.6^{2}}=19$ (μm)

### 2.3. MWR Payload Development

**Figure 2:**
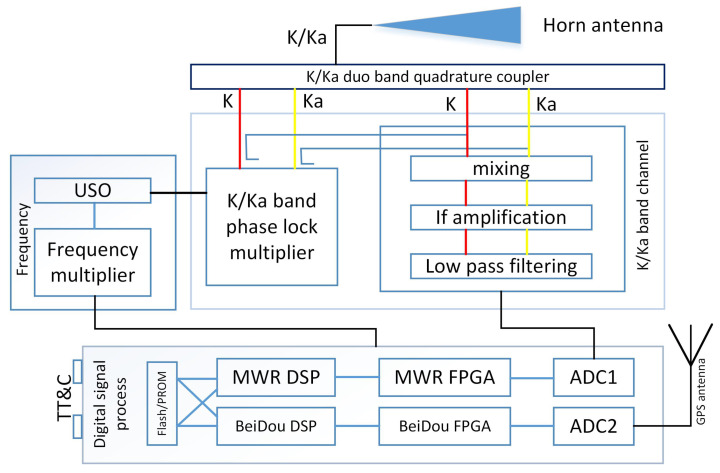
Block schematic of the microwave ranging (MWR) system, mainly including a K/Ka horn transceiving antenna with quadrature coupler, frequency reference unit, K/Ka-band microwave channel, and digital signal processing platform.

The MWR system payload has been carefully designed and developed, with the diagram of one spacecraft shown in Figure 2. Several things have to be determined during the design phase, including detailed system composition, frequency planning, link budget analysis, system measurement error analysis, antenna polarization mode and optimization, high-precision time synchronization, on-ground test and verification, etc.

Currently, MWR system payload prototypes have been developed that consist of two sets of identical equipment, mainly including (a) a K/Ka-band transceiving antenna: a single corrugated horn antenna that transmits–receives K/Ka-band dual-frequency microwave signals; (b) a frequency reference unit: an ultra-stable oscillator (USO) is used as the frequency reference source of the whole system; (c) a K/Ka-band microwave channel: up-converts the reference frequency to transmission carrier frequency (TCF) and down-converts the TCF received from another satellite to a single-carrier signal that completes the amplification process and reception mixing; and (d) a digital signal processing unit: conducts sampling and digital processing of the down-converted K/Ka-band carrier phase signal and BeiDou signal to complete the high-precision carrier phase extraction. Here, we provide a brief introduction of these four components, as:

#### 2.3.1. Antenna Scheme Design

**Figure 3:**
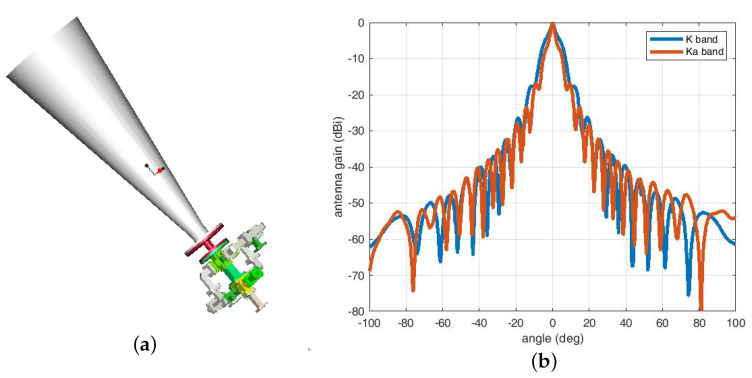
Illustration of the MWR transceiving antenna. (**a**) Three-dimensional antenna structure design including corrugated horn and K/Ka dual-polarization quadrature coupler. (**b**) Test results of the antenna pattern (radiation angle deviation from line-of-sight (LOS) direction vs. antenna gain) for both K and Ka bands.

The MWR antenna is composed of a K/Ka dual-frequency corrugated horn and a dual polarization quadrature coupler, as shown in Figure 3 below. The antenna was specially designed using a horn configuration, which has the advantages of small volume, compact connection with feed source, high-precision installation accuracy, and small thermal deformation (smaller thermal deformation of horn antennas made of invar steel). The disadvantage is that it is difficult to achieve high gain performance. Fortunately, this was finally solved by choosing the appropriate size of the corrugated horn, with a huge number of simulations and calculations.

#### 2.3.2. Frequency Reference Unit

The frequency reference units include an USO and a frequency multiplier, which provides a frequency reference for the microwave channel, signal processing unit of the MWR, and the BeiDou receiver.

The USO is achieved by a high-stability/low-phase-noise constant-temperature oscillator design, which includes (1) an oscillator circuit and (2) a temperature control circuit. The oscillator circuit is used in order to realize low phase noise function, and includes the main Pierce oscillator circuit, amplification, a spectrum purity filter, and a power supply circuit. A continuous temperature control circuit is adopted, since it guarantees the best short-term oscillator stability (less than $1\times 10^{-10}$/s) and temperature frequency characteristics (−40${}^{\circ }$C∼+80 ${}^{\circ }$C).

Frequency multipliers provide double and eight multiplier reference frequency with a power amplification, distribution, multiplication, and isolation amplification process.

#### 2.3.3. Microwave Channel

The differential frequency phase measurement method is adopted in order to achieve high-precision phase measurement in the MWR system. The phase measurement frequency of the Ka band is designed as 600 kHz. By making the reference frequency source of the USO from two spacecraft differ by 66 Hz, the carrier phase measurement can be completed with identical MWR equipment on-board the two spacecraft. The method uses the local transmitting carrier as the local oscillator for mixing, down-converts the receiving carrier, directly generates a phase measuring frequency point of about 600 kHz (i.e., the direct mixing technology of the transmitting carrier), and then sends it to the digital signal processing unit for carrier phase measurement.

The MWR microwave channel consists of an RF module, a local oscillator, and a secondary power supply. (a) The RF module uses a local oscillator signal to convert the 32.7 GHz RF signal to an 600.098 kHz intermediate frequency (IF) signal with a certain gain. The RF module gain can be adjusted by telecontrol command, with gain state parameters telemetry output. (b) The local oscillator uses the input reference signal in order to generate the local oscillator signal for RF module frequency down-conversion. (c) The secondary power provides the required secondary voltage for the RF module, and it controls the ON/OFF switch of the equipment through the telecontrol command, and it provides telemetry information on the switch state.

Fundamental wave mixing is adopted for the RF module’s down-conversion, which has the advantages of low frequency conversion loss (usually less than 10 dB) and low noise coefficient. Moreover, it can better suppress high-order mixing by-products when a single-balance or double-balance method is used. The central frequency of the fundamental mixing local oscillator signal is chosen as 32.7 GHz, according to the frequency relationship between input and output signals.

#### 2.3.4. Signal Processing Hardware Platform

The digital signal processing unit is the most important in the whole MWR system, since it processes both K/Ka dual-frequency signals and BeiDou dual-frequency navigation signals in high quality. New-generation BeiDou III B1C/B2a navigation signals are chosen, and dual-frequency receiver development is adopted, with the aim of providing high-precision time synchronization between satellites to the less than 0.3 ns that is required by MWR DOWR measurement.

**Figure 4:**
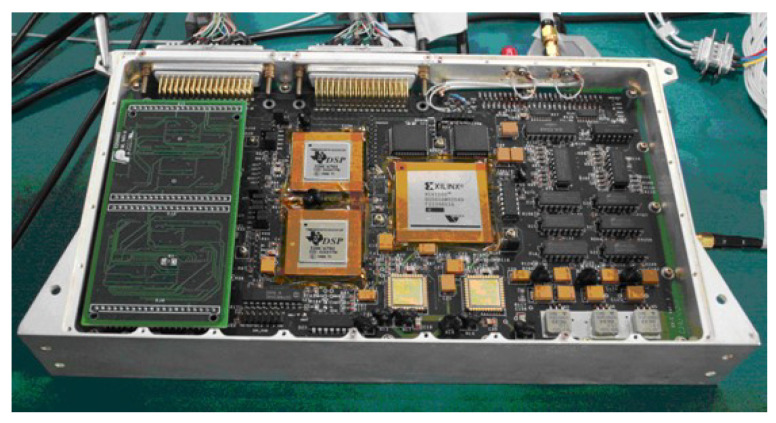
Signal processing hardware platform, including the K/Ka-band dual-frequency MWR processing module, BeiDou III B1C/B2a dual-frequency navigation processing module, and external interface module.

In order to ensure the time synchronization accuracy of 0.3 ns that is required by MWR, the dual-frequency navigation receiver is integrated with a K/Ka signal processing hardware platform that shares a unique USO frequency source. The hardware platform includes the MWR measurement processing module, the BeiDou measurement processing module, and the external interface module, as shown in Figure 4.

The ADC1 component of the hardware platform, as shown in Figure 2, is composed of two dual-channel analog-to-digital convertor (ADC) chips, which conduct the analog-to-digital conversion of K/Ka-band IF signals. The digitized K/Ka-band signals are sent to an FPGA for digital signal correlation processing to obtain the original measurement. The digital signal processor (DSP) completes the on-board relative ranging calculation, channel control, and data packaging to the GNC computer and TT&C external interface. Similar to the MWR process, ADC2 completes the analog-to-digital conversion of L band BeiDou B1C/B2a signals, sending to the BeiDou FPGA for digital signal correlation and original measurement processing. The BeiDou DSP completes the functions of high-precision time synchronization, channel control, and navigation positioning solution.

## 3. Relative Dynamics for Spacecraft Formation

Unlike the traditional relative orbit motion equation expressed in the directions *x*: radial, *y*: in-track and *z*: cross-track (RIC), with the origin located in the master spacecraft [17,18], here we use a radar system that was suggested by Eggleston and Dunning [19] by variables transformation as:

(11) x=ϱcosϕcosθ

(12) y=ϱcosϕsinθ

(13) z=ϱsinϕ

where ϱ denotes the relative distance between the formation spacecrafts, and θ and ϕ are the azimuth and relative elevation angle. The relative motion equations can finally be transformed to [20]:

(14) $$\ddot{\eta }=f\left(\eta ,\dot{\eta },t\right)+G\left(\eta \right)w$$

where $\eta ={\left[\varrho \;\;\theta \;\;\phi \right]}^{T}$, $w={\left[w_{\varrho }\;\;w_{\theta }\;\;w_{\phi }\right]}^{T}$, $f=\{f_{\varrho }\;\;f_{\theta }\;\;f_{\phi }\}$ with

(15) $$\begin{array}{cc} f_{\varrho }\left(\eta ,\dot{\eta },t\right)= & \left(\omega^{2}+2\omega \dot{\theta }+{\dot{\theta }}^{2}\right)\varrho \cos^{2}\phi +\varrho {\dot{\phi }}^{2}\\ \; & -\frac{\mu (r\cos\theta \cos\phi +\varrho )}{{\left[r^{2}+\varrho^{2}+2r\varrho \cos\theta \cos\phi \right]}^{\frac{3}{2}}}+\frac{\mu }{r^{2}}\cos\theta \cos\phi \end{array}$$

(16) $$\begin{array}{cc} f_{\theta }\left(\eta ,\dot{\eta },t\right)= & 2\left(\omega +\dot{\theta }\right)\dot{\phi }\tan\phi -\dot{\omega }-2\left(\omega +\dot{\theta }\right)\frac{\dot{\varrho }}{\varrho }\\ \; & +\frac{\mu (r\sin\theta \sec\phi )}{\varrho {\left[r^{2}+\varrho^{2}+2r\varrho \cos\theta \cos\phi \right]}^{\frac{3}{2}}}-\frac{\mu }{r^{2}\varrho }\sin\theta \sec\phi \end{array}$$

(17) $$\begin{array}{cc} f_{\phi }\left(\eta ,\dot{\eta },t\right)= & -\frac{1}{2}{\left(\omega +\dot{\theta }\right)}^{2}-\frac{2\dot{\varrho }\dot{\phi }}{\varrho }-\frac{\mu }{r^{2}\varrho }\cos\theta \sin\phi \\ \; & +\frac{\mu r\cos\theta \sin\phi }{\varrho {\left[r^{2}+\varrho^{2}+2r\varrho \cos\theta \cos\phi \right]}^{\frac{3}{2}}} \end{array}$$

The denotations of variables/matrix μ,r,ω,G(η), and w in (14)–(17) can be found in [20], and are not explained here for brevity. For the purpose of real formation simulation, the master and slave spacecrafts are separately propagated in an inertial frame, and the relative position and velocity are computed with differences and transformed into the master RIC frame and radar system in (11)–(17), considered to be the real orbit values. Gravitational and non-gravitational accelerations are considered, with the models shown in Table 1, as:

## 4. Mission Orbit and Relative Navigation Filter

### 4.1. Real Formation Mission Orbit

The formation flying orbit analyzed here, quite similar to that in PROBA-3, is a virtual structure SFF mission that will perform in HEO, which we previously referred to as the HEFFD mission. The HEFFD mission is initially designed as an on-orbit development, verification, and validation platform for multiple task forces. The main objective is to test guidance, navigation, and control (GNC) technology in space flight, especially for accurate ranging metrology units and high-performance propulsion systems that enable fine formation maneuvers.

The HEFFD mission orbit design is a trade-off analysis process that considers the requirements of: (a) high altitude during the formation flying stage since it is a low-gravity-gradient environment; (b) simple Sun–Earth geometry when SFF during Solar observation phase; (c) launch capability; and (d) ground station location.

Here, we provide the baseline orbit for the HEFFD mission in Table 2 as:

and the slave spacecraft is supposed to be performing follow-on flight relative to the master spacecraft, with a minimal distance of 10 km around apogee.

Some preliminary design parameters of master/slave spacecrafts include: mass: 200 kg/400 kg; reference area: 1.81 m${}^{2}$/3.34 m${}^{2}$; dimensional drag coefficient: 1.29/1.5; dimensional solar radiation pressure coefficient: 1.2/3.27 [21,22].

**Figure 5:**
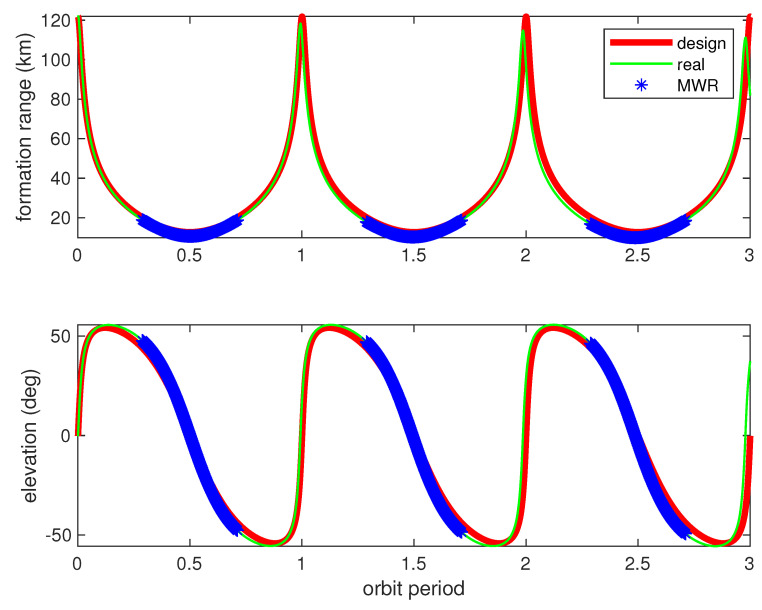
Formation range and elevation angles (design and real values). The red lines denote the designed spacecraft formation flying (SFF) relative range (**top**) and relative elevation angles (**bottom**) during three orbit periods, and the green lines denote the same values of SFF in real orbit perturbations. The blue lines denote the mission operation orbit during apogee for precise SFF guidance, navigation, and control (GNC) technology verification.

**Figure 6:**
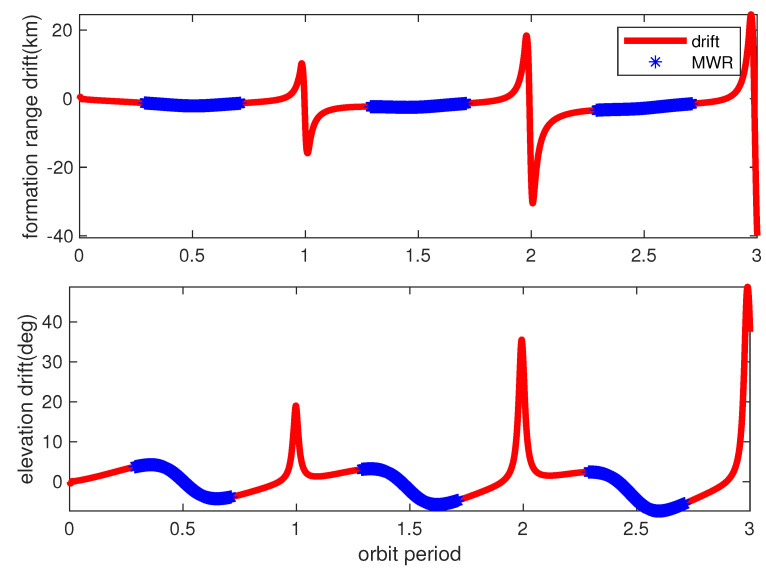
Formation range and elevation angle bias. The red lines represent the bias of real formation range drift from the design values (**top**) and the elevation angle drift (**bottom**). The blue lines denote the MWR equipment operation time during apogee.

Figure 5 and Figure 6 provide the relative range and elevation angle values for three orbit periods. Clearly, the orbit calculation starts from perigee with a relative formation distance of about 120 km, and gradually approaches to a minimum of about 10 km, which is suitable for mission operation. The bolded red lines in Figure 5 demonstrate the relative orbit values of range and elevation angles, while using dynamic equations of the radar model (11)–(17). The azimuth angle values are not shown here, since the co-orbit formation configuration is used in this simulation. Moreover, the green lines in Figure 5 show the relative range and elevation values by using the propagated perturbation orbit dynamicsmodel in Table 1. The results illustrate that the real perturbed relative formation orbit drifted quickly from the radar model during simulation, and Figure 6 illustrates the drift bias within three orbit periods.

According to Figure 5 and Figure 6, the relative orbit dramatically drifted. The formation ranging distance drifted gradually near each perigee arc, almost reached −40 km at the end of the third orbit time, and converged quickly during each apogee arc. The formation mission configuration can be carefully designed initially, considering detailed orbit perturbations. However, a sophisticated relative model is not suitable for navigation filter calculation due to the huge on-board computation burden for real space missions. Finally, by careful consideration, apogee arcs were chosen as the mission operation orbit, which is used for high-precision formation GNC technology verification. The mission apogee arcs, occupying about half of the orbit period time, are clearly shown in Figure 5 and Figure 6, using bolded blue star dots. The relative navigation algorithm has to be carefully designed, considering real orbit drift and model uncertainty.

### 4.2. Relative Navigation Using Adaptive Kalman Filter

According to the relative orbit model (11)–(17), the class of systems considered here is given as

(18) $$\begin{array}{cc} x_{k} & =f\left(x_{k-1},u_{k-1}\right)+Gw_{k-1}\\ y_{k} & =h\left(x_{k}\right)+v_{k} \end{array}$$

where $x_{k}\in R^{n}$ denotes the state vector and $u_{k}\in R^{m}$ denotes the control vector. The output vector is $y_{k}\in R^{p}$ and the non-linear model is $f(x_{k-1},u_{k-1})$. The process/measurement noise $w_{k}$ and $v_{k}$ are assumed to be independent, identically distributed Gaussian random variables with

(19) $$w_{k}∼\;N\left(0,Q_{k}\right)\;,\;\;\;v_{k}∼\;N\left(0,R_{k}\right)$$

The traditional Kalman filter provides the optimal estimation of states by using a recursive scheme of propagating states and measurement update. The filter optimally blends the prior state and information update through a gain matrix which balances uncertainty in both the measurements and dynamics model.

However, for HEO SFF missions, the real relative orbit drifted gradually away from the designed model, as in Figure 6. The orbit drift is inevitable due to the unknown process noise from perturbations. Some adaptive approaches have to be used for the on-line estimation of process noise, which will increase the accuracy of the predicted state error covariance matrix and increase the stability of the filter.

Here, we use a recursive adaptive algorithm dealing with process noise uncertainty by minimizing a function in real time, which is determined by the difference of residual covariance computed by the filter and residual sequence generated by the filter. The adaptive approach can be summarized as follows:

Suppose that we have the predicted state error covariance matrix $\bar{P}\left(k\right)$, then ${\bar{P}}_{y}\left(k\right)=H\bar{P}\left(k\right)H^{T}+R\left(k\right)$ is the predicted output covariance matrix. The adaptive Kalman filter for unknown process noise is used to minimize the following criterion function

(20) $$S_{k}=\mathrm{tr}\left(\Delta {\bar{P}}_{y}^{2}\left(k\right)\right)=\mathrm{tr}\left[{\left({\bar{P}}_{y}\left(k\right)-{\bar{P}}_{y}^{0}\left(k\right)\right)}^{2}\right]$$

where ${\bar{P}}_{y}^{0}\left(k\right)$ is the unbiased estimation of ${\bar{P}}_{y}\left(k\right)$. The traditional Massachusetts Institute of Technology (MIT) rule is used here to derive the adaptive law, which is a popular method for the on-line estimation of unknown parameters in a control system [23,24]. The solution of criterion function (20), including the process of negative gradient, discretization, and recursion for process noise update. These adaptive processes have been successfully used in HEO SFF, which are not shown here for brevity [25].

## 5. Test and Simulation

**Figure 7:**
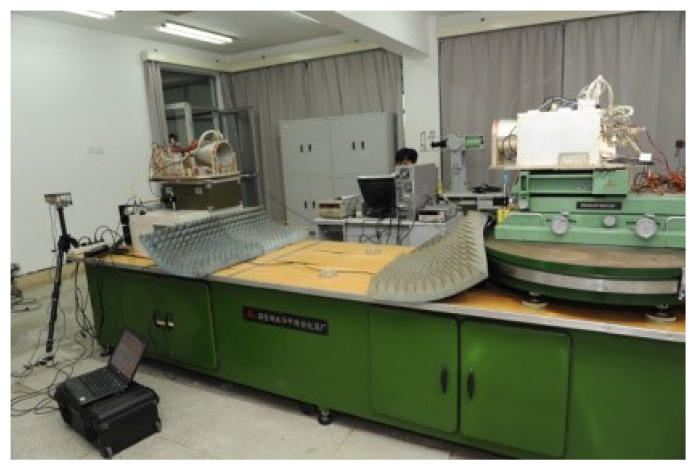
Six degree of freedom moving platform that achieved micron-level accuracy in position and an accuracy of milliarcseconds in attitude orientation.

Extensive tests of the MWR payload were done in the laboratory environment, conducted in a precise moving platform, as shown in Figure 7. The moving platform can perform six degree of freedom (6-DOF) movement with an accuracy of microns in position and milliarcseconds in attitude orientation. The test work was divided into two stages: first, the assessment of MWR ranging accuracy by simply using K-band microwave signal processing, through transmitting and receiving. The output of this stage is the knowledge of the best accuracy that MWR can provide, which is considered as a fundamental specification for the next step. The second stage is hardware-in-the-loop (HIL) simulation. To conduct an in-depth analysis of the relative navigation filter performance using real MWR measurement equipment, an HIL simulation was run with all of the payloads fixed in the 6-DOF platform that will be deployed on-board in future SFF missions. Relative navigation errors that are provided by different measurement payloads and different filter algorithms were obtained at this stage, and the trade-off analysis for SFF mission reward and measurement capability was determined.

### 5.1. Assessment of MWR Ranging Accuracy

**Figure 8:**
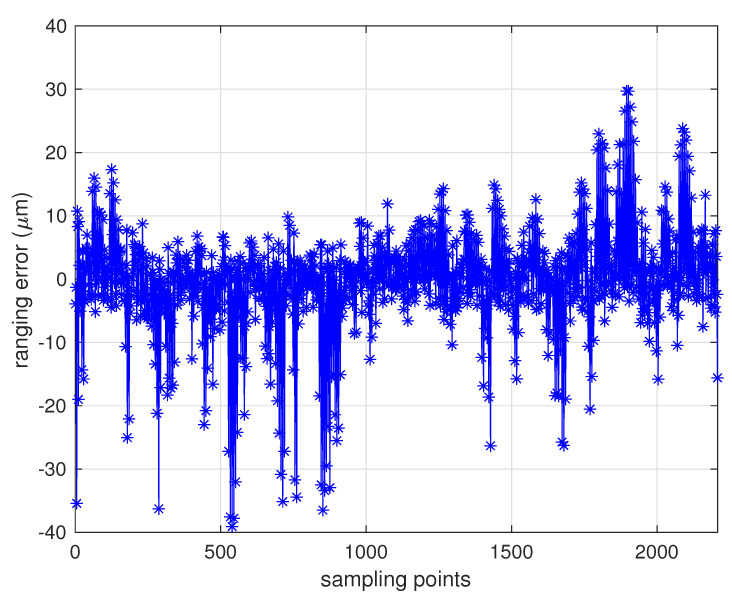
MWR ranging errors of 2400 samples, with data collected from a 6-DOF moving platform under a relative longitudinal velocity of 5 μm/s. The ranging errors were less than 40 μm during the test.

**Figure 9:**
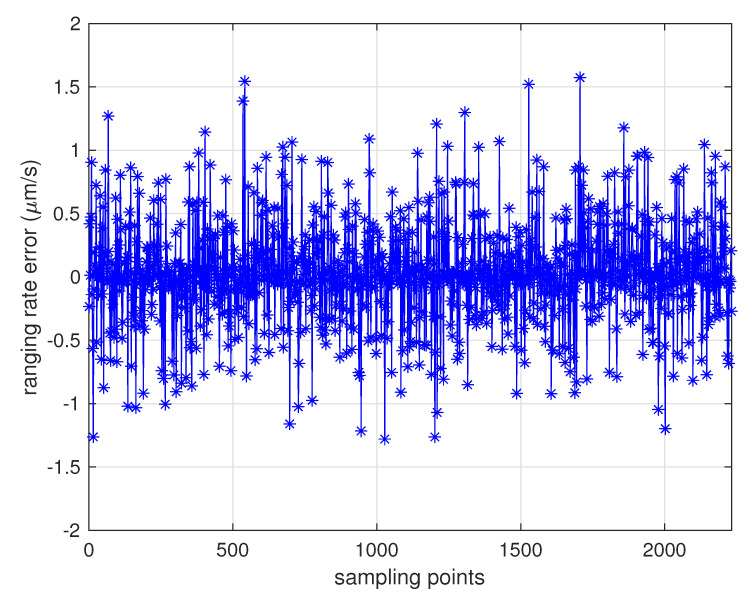
MWR ranging rate errors of 2400 samples, with data collected from a 6-DOF moving platform under a relative longitudinal velocity of 5 μm/s. The ranging rate errors were less than 1.6 μm/s during the test.

With extensive tests of MWR that were conducted in the 6-DOF platform, the results verified the micron-level high-accuracy specifications that could be applied to real space formation missions in the future. Table 3 provides the primary design parameters of the whole ranging system, with a relative distance of 100 km as an example. Figure 8 and Figure 9 provide the real ranging test results from the 6-DOF moving platform under a stable longitudinal velocity of 5 μm/s. The ranging error (the differential between real measurement data from MWR and optical moving platform sensor) was less than 40 μm during the test process, and 1.6 μm/s range rate error was obtained.

### 5.2. Hardware-in-the-Loop Simulation for SFF Relative Navigation

A hardware-in-the-loop (HIL) simulation was conducted, with all of the deployed payloads fixed in the 6-DOF platform, aiming for an in-depth analysis of the relative navigation filter performance using real MWR equipment.

When considering the sharp orbit drift during perigee time, mission designers proposed a mature S-band ranging (SBR) equipment, in addition to a BeiDou dual-frequency receiver, as a backup payload for both relative measurement data transfer and TT&C payloads. SBR is a reliable medium-accuracy measurement payload that operates at the amateur frequency of 2450 MHz ± 50 Hz. The ranging accuracy of SBR is 1 m(1σ) in pseudo-noise (PN) code measurement mode, 0.01 m(1σ) in carrier phase measurement mode, and the data rate achieved a maximum of 1 Mbit/s. The whole ranging process during the HEFFD mission is scheduled as follows: (1) The BeiDou B1C/B2a dual-frequency receiver is operated during the whole flight, which provides relative carrier phase differential measurement of centimeter-level accuracy, and provides a synchronized MWR time tag. (2) The SBR payload is used for reliable medium-accuracy distance measurement and data transfer, which is arranged to operate during the whole flight time. (3) The relative ranging values from the BeiDou receiver and SBR are simply optimally fused at the output level. This will be regarded as baseline formation distance data in HEFFD mission. (4) The MWR payload is especially developed for the HEFFD mission, and it will only be used during the apogee orbit period for submillimeter-level ranging and precise GNC technology validation. (5) Dual-frequency interference optical ranging payload is also used with a ranging accuracy of 1 μm(1σ), which provides the reference distance data for the validation process.

**Figure 10:**
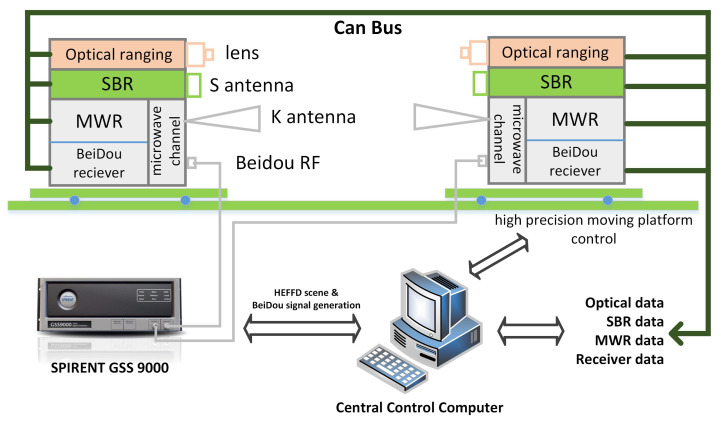
Block diagram of the simulation platform, mainly including a high-precision 6-DOF moving platform, optical/S-band ranging (SBR)/MWR/BeiDou payloads, a SPIRENT GSS9000 simulator, central control computer, and standard CAN bus.

The platform was precisely installed with transmitting/receiving payloads and antennas of SBR, MWR, and optical equipment, with a guaranteed accuracy of a fixed position deviation within two microns. Figure 10 demonstrates the block diagram of the whole simulation platform used in this paper. The whole simulation system was coordinated by a central control computer, which conducted the HEFFD mission formation scenario design, high-precision moving platform control, payload operation, data collection, and analysis. Note that a state-of-the-art high-fidelity SPIRENT GSS9000 simulator was used for the generation of BeiDou B1C/B2a signals and real HEFFD mission scene. Dual-frequency BeiDou receivers from both formation spacecrafts were connected directly to the simulator for the purpose of differential carrier phase measurement and precise time synchronization with an accuracy of 0.3 ns(1σ).

Some of the parameters used in this simulation were as follows: initial date of simulation: 8 April 2021, 14:15:00 (GMT+08:00), sample time interval: 1 s. The spectral densities of the process noise components $w_{x},w_{y},w_{z}$ in Equation (19), which are each given by $\sqrt{5}\times 10^{-11}$ m/s${}^{3/2}$[20]. The individual standard deviations for initial states η and $\dot{\eta }$ were 0.01 km for ϱ, 0.1 deg for θ, 0.1 deg for ϕ, $1\times 10^{-4}$ km/s for $\dot{\varrho }\left(0\right)$, 0.01 deg/s for $\dot{\theta }\left(0\right)$, and 0.01 deg/s for $\dot{\phi }\left(0\right)$, 0.05 and 0.05 deg/s${}^{2}$ for leading orbit angular velocity and its rate, and 50 m and 0.01 m/s for the leading orbit radius and its rate.

**Figure 11:**
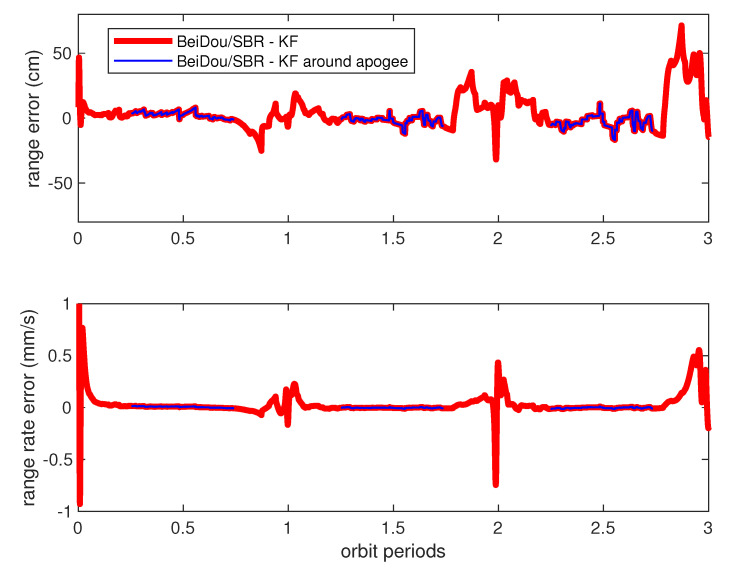
Range and range rate estimation errors from BeiDou/SBR equipment. The red lines denote the estimation errors, and the short blue lines denote the same data during apogee time. KF: traditional Kalman filter.

Figure 11 illustrates the estimation error (the differential of estimated values and real values from the model in Table 1) of HEFFD’s relative range and range rate by using BeiDou/SBR measurement and the traditional Kalman filter algorithm. The performance in this situation was not good, as the estimation error was divergent gradually over time, especially during perigee periods (bolded red lines in Figure 11), and converged slightly near apogee (blue lines in Figure 11). The range estimation error was up to 80 cm around perigee, and reduced to about 10 cm during apogee flight. The range rate error achieved a maximum of 1 mm/s during perigee, and 0.03 mm/s during apogee. Similar estimation performance of elevation, azimuth angle, and their rates was obtained, which will be provided later in detail.

**Figure 12:**
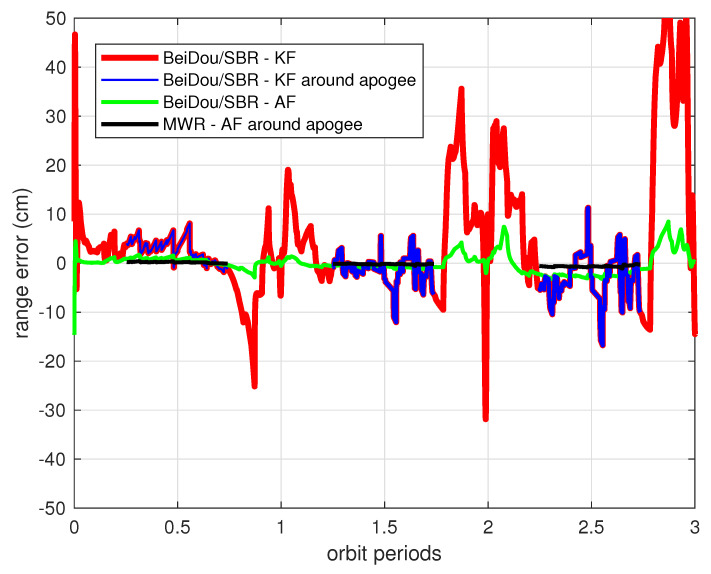
Relative range estimation errors from different payloads and filters. The red line shows the relative range estimation errors of BeiDou/SBR payload using the traditional Kalman filter algorithm (the same data as during apogee time in the blue lines); the green line represents the estimation errors of the BeiDou/SBR payload using the adaptive filter (AF); the black lines show the estimation errors of the MWR payload using the adaptive filter during apogee time.

**Figure 13:**
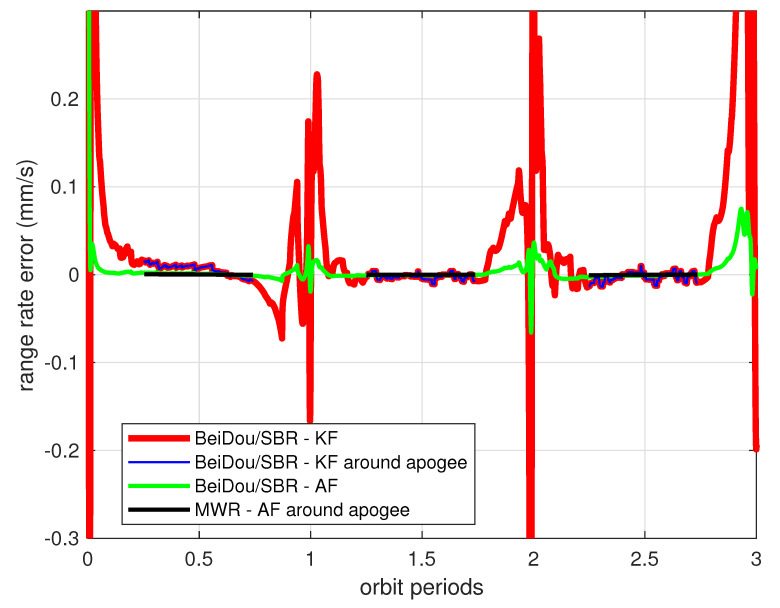
Relative range rate estimation errors from different payloads and filters (refer to Figure 12 for the denotation of color lines).

Clearly, this is not an optimal result for engineering application. The reason for the filter divergence is that the formation dynamic model used in the filter (i.e., radar model) is not sufficiently accurate for the prediction of states through recursive calculation at each sampling time. The process noise values of $w_{x},w_{y},w_{z}$ change consistently according to the orbit perturbations, and need to be adjusted through an adaptive approach from measurement updates. For the purpose of comparison, the data analyzed in Figure 11 are redrawn in Figure 12 and Figure 13 with new results. The green line in Figure 12 demonstrates the relative ranging errors while using the adaptive filter introduced in Section 4, from BeiDou/SBR equipment. It can be seen that the estimation accuracy notably improved during both perigee and apogee orbit time, as a result of using the process noise adaptive filter algorithm. The range estimation error was less than 10 cm around perigee, and reduced to 1 cm during apogee. The range rate estimation errors were also significantly reduced, as can be seen in Figure 13, achieving less than 15 μm/s around apogee.

However, centimeter-level ranging accuracy is not sufficient for the real HEFFD mission. The black lines in Figure 12 and Figure 13 illustrate the MWR results using the adaptive approach during apogee periods in the simulation. With data collection, statistics, and analysis after simulation, the final accuracy of MWR was 35 μm in range and 8.5 μm/s in range rate. Table 4 provides the maximum estimation error and root mean square (RMS) error during three orbiting periods, for the full states, using different relative filter algorithms and ranging equipment. The results clearly demonstrate the effectiveness of an adaptive filter that incorporates process noise uncertainty, and submillimeter-level ranging accuracy for formation flight in HEO using MWR technology.

## 6. Conclusions

In this study, we presented a novel submillimeter-level accuracy K-band microwave ranging (MWR) payload, used for the on-orbit demonstration of precise ranging technology for a future HEO SFF mission, which we have named the highly elliptical formation flying demonstration (HEFFD) mission. The MWR uses DOWR microwave phase accumulation, and details on the development of MWR are introduced, including a K/Ka-band dual-frequency corrugated horn antenna, frequency reference unit, microwave channel, and signal processing platform. Relative orbit models with perturbations and an improved adaptive filter algorithm that incorporates process noise uncertainty are presented, and are used for the SFF relative navigation system design.

The MWR equipment was tested in two stages: an assessment of the ranging accuracy, and an HIL simulation within the SFF GNC system. First, the MWR payload was fixed in a high-precision 6-DOF moving platform and extensively tested under a relative velocity of 5 μm/s. The ranging errors were less than 40 μm and the range rate errors were 1.6 μm/s. Second, a complex HIL simulation system was constructed which incorporated an optical sensor, a BeiDou dual-frequency receiver, and SBR equipment, aiming to evaluate real relative navigation filter performance during the HEFFD mission. Our results indicate that the final MWR range estimation accuracy using the adaptive filter algorithm was less than 35 μm and it was 8.5 μm/s for the range rate, which clearly demonstrates the promising submillimeter-level performance for future HEO formation missions.

## Figures and Tables

**Table 1:** Accelerations of gravitational and non-gravitational models.

Items	Model
GA—the geopotential effect of the Earth	20th order and degree
GA—Sun and Moon gravities	high-precision DE405/LE405 planetary ephemerides model
GA—solid Earth tides	IERS Conventions 1996
GA—ocean tides	Center for Space Research 3.0 model
NGA—the atmospheric drag	NRLMSISE-00 empirical model
NGA—the solar radiation pressure	IERS Standards 1992

Note: GA: gravitational acceleration; NGA: non-gravitational acceleration.

**Table 2:** Highly elliptical formation flying demonstration (HEFFD) mission orbit parameters (master spacecraft).

Parameter	Value	Unit
Apogee altitude	60,530	km
Perigee altitude	600	km
Inclination	59	deg
Argument of perigee	42	deg
RAAN	25	deg
True anomaly	0	deg

**Table 3:** Parameters of MWR ranging payloads.

Items	Symbol	Unit	K Band	Ka Band
Frequency	*f*	GHz	24.517002	32.692746
Transmitting power	Ptx	W	10	10
Transmitting power	Ptx	dBW	10	10
Transmitting feed loss	Lftx	dB	0.5	0.5
Transmit antenna gain	Gtx	dBi	16	16
Effective isotropic radiated power	EIRP	dBW	25.5	25.5
Space distance	*d*	km	100	100
Flux density	Fd	dBW/m${}^{2}$	−85.49209857	−85.49209857
Space propagationloss	Ld	dB	−160.2311193	−162.7308
Power level of receiving antenna	Prxa	dBW	−134.7311193	−137.2308
Receiving antenna gain	Grx	dBi	15	15
Polarization mismatch loss	Lpol	dB	1	1
Antenna gain of receiving system port	Grxs	dBi	14	14
Receiving feed loss	Lfrx	dB	0.5	0.5
Power level of receiver input port	Prxi	dBm	−91.23111929	−93.73080004
Noise temperature of receiving antenna	Ta	K	150	150
Temperature of receiving feeder	Tf	K	290	290
Receiver noise factor	Rf	dB	10	10
Receiver noise temperature	Terx	K	228	228
Receiver noise temperature	Terx	dBK	23.57934847	23.57934847
ENT of Receiver port	Td	K	393.2248687	393.2248687
ENT of Receiver port	Td	dBK	25.94640976	25.94640976
G/T value of Receiver port	G/T	dB/K	−12.44640976	−12.44640976
ENT at the receiver system port	Terxs	K	441.2055593	441.2055593
ENT at the receiver system port	Terxs	dBK	26.44640976	26.44640976
G/T value of receiver system port	G/T	dB/K	−12.44640976	−12.44640976
Carrier-to-noise ratio	$C/N_{0}$	dB-Hz	80.82247095	78.3227902

**Table 4:** Relative navigation estimation error during simulation.

Parameter	Unit		KF-BeiDou/SBR*		AF-BeiDou/SBR*		AF-MWR	
			Perigee	Apogee	Perigee	Apogee	Perigee	Apogee
range	μm	ME*	$7.8\times 10^{5}$	$8.65\times 10^{4}$	$9.12\times 10^{4}$	$1.05\times 10^{4}$	/	$3.5\times 10^{1}$
		RMS	$3.02\times 10^{5}$	$5.65\times 10^{4}$	$3.64\times 10^{4}$	$8.27\times 10^{3}$	/	9.50
range rate	μm/s	ME	$1\times 10^{3}$	$3\times 10^{1}$	$9\times 10^{1}$	$1.5\times 10^{1}$	/	8.5
		RMS	$5.91\times 10^{2}$	$2.54\times 10^{1}$	$4.51\times 10^{1}$	9.8	/	2.6
elevation angle	deg	ME	$5\times 10^{-2}$	$5\times 10^{-2}$	$4\times 10^{-3}$	$3\times 10^{-3}$	/	$1.4\times 10^{-3}$
		RMS	$4.21\times 10^{-2}$	$3.4\times 10^{-2}$	$3.1\times 10^{-3}$	$2.2\times 10^{-3}$	/	$9\times 10^{-4}$
elevation rate	deg/s	ME	$1.9\times 10^{-3}$	$1.7\times 10^{-3}$	$9.2\times 10^{-4}$	$7\times 10^{-4}$	/	$1.2\times 10^{-4}$
		RMS	$1.8\times 10^{-3}$	$1.54\times 10^{-3}$	$8.14\times 10^{-4}$	$5.22\times 10^{-4}$	/	$5.24\times 10^{-5}$
azimuth angle	deg	ME	$8\times 10^{-2}$	$7\times 10^{-2}$	$4\times 10^{-3}$	$4\times 10^{-3}$	/	$1.5\times 10^{-3}$
		RMS	$6.1\times 10^{-2}$	$4.8\times 10^{-2}$	$3.02\times 10^{-3}$	$2.6\times 10^{-3}$	/	$1.1\times 10^{-3}$
azimuth rate	deg/s	ME	$1.9\times 10^{-3}$	$1.9\times 10^{-3}$	$8.6\times 10^{-4}$	$8\times 10^{-4}$	/	$1.3\times 10^{-4}$
		RMS	$1.62\times 10^{-3}$	$1.59\times 10^{-3}$	$7.03\times 10^{-4}$	$6.21\times 10^{-4}$	/	$8.16\times 10^{-5}$

* Note: KF: typical Kalman filter; AF: adaptive filter; ME: maximum error.
